# An end-to-end data analysis framework for real-time detection and source identification of pollution events via e-nose networks

**DOI:** 10.1007/s00216-025-06014-8

**Published:** 2025-07-24

**Authors:** Mahsa Akbari Lakeh, Simon Bootsma, Ralph van Nellestijn, Gerjen H. Tinnevelt, Jeroen J. Jansen

**Affiliations:** 1 Institute for Molecules and Materials (Analytical Chemistry), Radboud University, Nijmegen, 6500 GL 9010, The Netherlands; 2 Comon Invent B.V, Delft, Burgemeestersrand 198a, 2625 NZ The Netherlands

**Keywords:** Ambient air quality monitoring, Electronic noses (e-noses) network, Pollution source apportionment, Spatiotemporal event detection, Emission event characterization

## Abstract

Ambient air pollution contributes to an estimated 4.2 million premature deaths worldwide each year, underscoring the imperative for advanced air quality management tools. In heavily industrialized regions, diffuse fugitive leaks and concentrated stack emissions threaten public health and ecosystems, making real-time monitoring and accurate source apportionment indispensable. To address these challenges, we introduce an end-to-end data analysis framework that employs a distributed network of low-cost electronic noses (e-noses) to deliver spatially and temporally resolved emission monitoring. The real-time, high-resolution outputs of e-noses are deconvoluted into discrete emission events using a chemometric pipeline including principal component analysis (PCA), hierarchical cluster analysis (HCA), and multivariate curve resolution-alternating least squares (MCR-ALS). Each event is then characterized within a 5W attribution schema, which identifies what anomaly was detected, when, and where it occurred, why it arose, and who is responsible for mitigation, thereby creating a searchable database of pollution incidents. By combining real-time, low-cost sensing with robust multivariate analysis and stakeholder-centered reporting, this framework enables transparent emission accountability. In turn, it supports rapid mitigation responses and strengthens regulatory compliance in complex industrial regions.

**Graphical Abstract:**

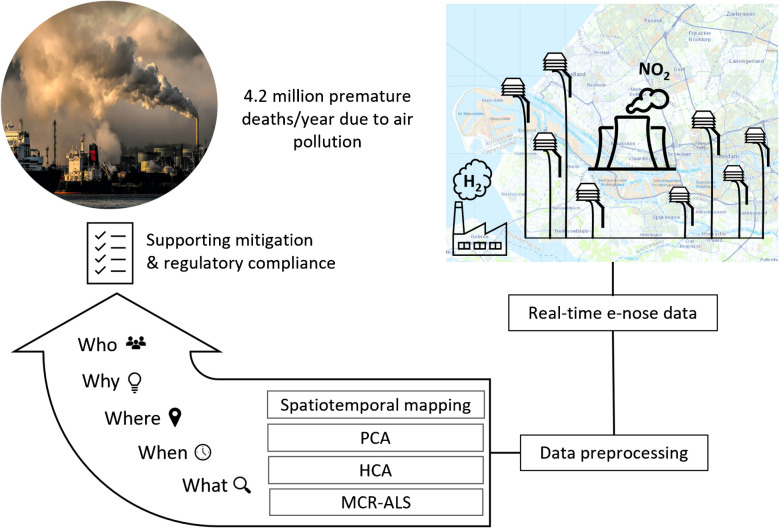

**Supplementary Information:**

The online version contains supplementary material available at 10.1007/s00216-025-06014-8.

## Introduction

Ambient air quality is a critical determinant of public health and environmental sustainability. The adverse effects of airborne pollutants on human well-being are well documented [1, 2], necessitating continuous and precise monitoring to detect and address pollution events before they escalate into public health crises. Conventional monitoring systems have largely relied on fixed-site stations that measure key pollutants such as nitrogen oxides (NOx), sulfur dioxide (SO_2_), carbon monoxide (CO), volatile organic compounds (VOCs), and ozone (O_3_). However, emerging research highlights that even gases typically considered benign, like hydrogen (H_2_), can have indirect effects by altering atmospheric chemistry and contributing to secondary pollutant formation, notably increasing ozone production [3].

Monitoring strategies are evolving beyond static concentration measurements to better account for dynamic parameters such as spatiotemporal variability [4]. This shift is driven by the inherently dynamic nature of atmospheric conditions and the rapid changes in industrial activities and urbanization, which require systems capable of capturing both pollutant emissions and their variability over space and time. Real-time, high-resolution data are essential for identifying pollutant peaks, trends, and anomalies that could indicate emerging environmental hazards. Equally important is the integration of meteorological data that govern pollutant dispersion and chemical transformations in the atmosphere.

The evolution of sensor technology has been central to the development of novel monitoring solutions. First studies in the early 1990 s established the foundational principles of electronic nose (e-nose) technology [5], and more recent reviews by [6] and [7] have highlighted the shift toward deploying low-cost, sensor-based networks capable of delivering high-resolution spatial and temporal data. Complementing this trend, the NAMUR Roadmap for Process Sensors [8] emphasizes the need for modular, intelligent, and digitally integrated sensors to support predictive maintenance. Despite this advance, many current systems fail to seamlessly integrate real-time digital data with robust source apportionment methodologies. Traditional approaches, which predominantly rely on concentration data registered at fixed monitoring sites, do not always capture the nuanced variations in pollutant profiles that arise from transient or localized emission events [9].

Electronic noses offer a promising solution to these challenges. By mimicking the human olfactory system through arrays of cross-reactive gas sensors, e-noses can detect a wide range of airborne analytes without necessitating individually selective sensors [10, 11]. Their compact design, low maintenance requirements, and cost-effectiveness make them ideal for large-scale deployment in both urban and industrial settings. Moreover, when coupled with advanced machine learning algorithms, e-nose networks can detect anomalies with high sensitivity and speed, while also converting raw sensor signals into actionable data that reflect discrete pollution events. This capability is essential for transforming quantitative measurements into clear, interpretable indicators that can support timely interventions and inform public–private dialogue on air quality improvement.

Beyond detection of emission anomalies, a significant challenge lies in differentiating emission risks to tailoring appropriate responses. Not every deviation in pollutant levels signals an urgent environmental threat, or is equally actionable. Routine emissions from controlled industrial processes, transient variations during startup or maintenance operations, and true pollution incidents such as chemical leaks or equipment malfunctions each require distinct management strategies. Current regulatory frameworks require monitoring systems to record emission levels, reliably identify the sources, and assess the associated risks [12]. Accurate source apportionment is thus critical for determining whether elevated emissions are attributable to known local sources or external passing plumes, which in turn dictates the urgency and nature of remedial actions.

To increase the Findability, Accessibility, Interoperability, and Reusability (FAIR) [13] of emission data for anomaly detection and mitigation, we propose a comprehensive framework structured around the 5Ws—*what*, *when*, *where*, *why*, and *who*—aiming to translate the voluminous data into discrete, quantified, and interpreted events. This rhetorical structure, advocated by Sloan [14] and rooted in classical inquiry [15], enables a systematic identification and contextualization of emission events. By framing the monitoring process within these five dimensions, our approach enhances detection accuracy while offering a nuanced, discretized context essential for informed decision-making across all aspects of an anomaly or emission event.

Building on this conceptual foundation, the framework classifies emission events into distinct categories such as routine releases, non-critical operational variations, and acute pollution incidents. This classification supports timely and effective responses from regulatory bodies and industry stakeholders. The objective of this study is to integrate an existing network of metal oxide semiconductor (MOS) e-noses with a novel, real-time, data-driven framework based on the 5 W structure. Unlike conventional source apportionment studies, which rely mostly on fixed-site concentration data [9], our approach leverages the multidimensional data captured by e-nose signals and contextualizes it within the 5 W framework to rapidly identify and classify emission events. This method enables detection of subtle variations in pollutant profiles and facilitates rapid source apportionment, transforming raw sensor data into discrete, context-rich events suitable for decision-making.

To illustrate the capabilities of this approach, we present a case study based on 24-h data collected from a segment of an e-nose network monitoring the Rotterdam port, a high-profile industrial hub with complex emission patterns. This study demonstrates how real-time data integration, chemometric analysis, and the 5 W framework together can reveal detailed spatial and temporal pollution patterns. To further support the interpretation of specific emission sources, regional air quality monitoring data were analyzed using the same data analysis methodology. The results underscore the potential for early anomaly detection and targeted response, while also showcasing the framework’s ability to distinguish between various emission sources in a complex industrial setting.

## Materials and methods

This section details the study area and data collection by the monitoring network (“Studyarea” and “Data collection” sections), the data pre-processing and anomaly detection methods (“Data pre-processing” and “Anomaly detection: what, and when” sections), and the source identification procedures, including multivariate analysis techniques (Sections “Source identification: where, why, and who”, “Principal component analysis (PCA) and hierarchical cluster analysis (HCA)”, and “Multivariate curve resolution-alternating least squares (MCR-ALS)”).

### Study area

This study was conducted in the Vondelingenplaat region within the Port of Rotterdam, a major industrial hub situated near the Maas River and key transportation routes. The area hosts extensive petrochemical, refining, and logistics operations. It is also located near residential neighborhoods including Pernis, Hoogvliet, and Vlaardingen, where community concerns over air quality have led to ongoing monitoring efforts. The Vondelingenplaat was selected for focused study due to its industrial density, logistical accessibility, and its representation of typical emission environments within the port.

### Data collection

A system of 22 e-noses (Comon Invent, Delft, The Netherlands) was employed to monitor ambient air quality in the Vondelingenplaat region. Each device is equipped with an array of four metal oxide (MOS) gas sensors, which do not quantify individual pollutants but respond broadly to reactive airborne chemical species present in the air. The e-noses are equipped with 4G modems and transmit data to a central server in real time, with a logging frequency of 1 min.

E-nose signals typically remain at background levels throughout the year, with elevated readings generally corresponding to permitted emissions or occasional pollution incidents. To evaluate the performance of the proposed data analysis framework, a 24-h period—from 12:00 PM on 25 January to 12:00 PM on 26 January 2023—was selected, during which at least two higher-than-normal emission events were detected in the target region.

In addition to e-nose data, real-time meteorological and air quality data from a nearby weather station and the Landelijk Meetnet Luchtkwaliteit (LML) monitoring network were used. The weather station data includes wind direction and speed, which were analyzed to investigate the influence of meteorological conditions on the dispersion and transport of air pollutants. The LML stations report concentrations of specific air pollutants, including nitrogen monoxide (NO), nitrogen dioxide (NO2), and particulate matter (PM10). To examine long-term air quality patterns in the vicinity of Vondelingenplaat, LML data collected in Schiedam during 2021 and 2022 was analyzed.

**Fig. 1:**
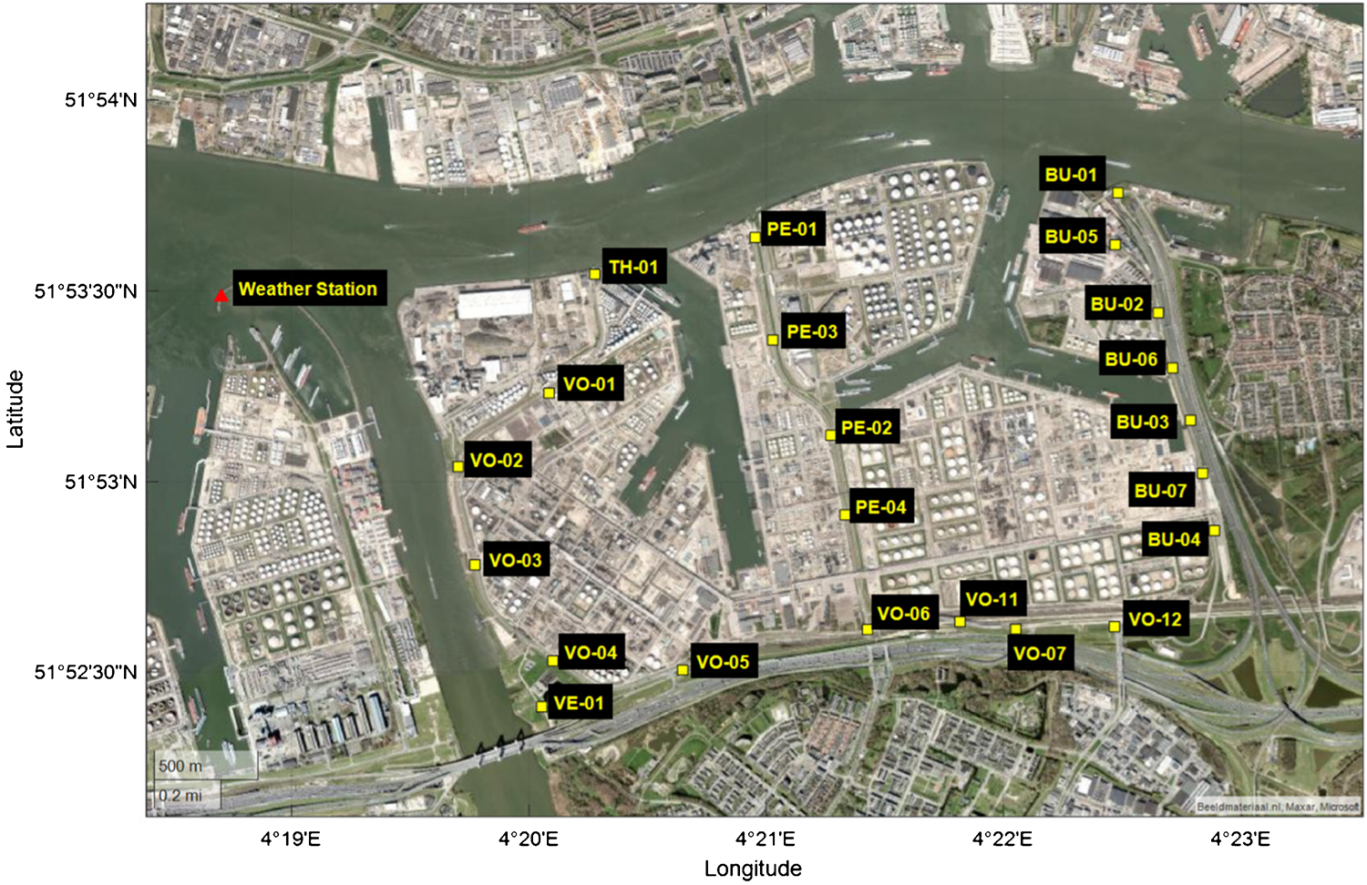
Geographic distribution of monitoring points and the weather station within the study area. This map illustrates various e-nose locations, identified by their respective labels, situated along waterways and industrial zones. Potential pollution sources, including vessels, industrial tanks, and highways, are prominent in the region. Additionally, residential areas are located near the study site, as depicted in the figure

Figure 1 presents a map of the Vondelingenplaat region, showing the location of installed e-noses, labeled with their assigned abbreviations, the nearby weather station, and potential pollution sources in the area. The monitored area is primarily industrial and located in close proximity to residential neighborhoods.

### Data pre-processing

The following pre-processing steps were undertaken to ensure that the e-nose data were clean, synchronized, and suitable for anomaly detection. The data collected from the e-nose network was converted from JSON to a MATLAB-readable format to facilitate subsequent analysis. Next, the desired time span for the selected e-noses was extracted from data. In the following step, the total signal of each e-nose was calculated by summing the signals from individual sensors within each e-nose over time. This total signal was then smoothed using a robust lowess approach [16], with a window length equal to 2% of the data length. To synchronize the signal from individual e-noses, which were logged at slightly different time points, mean filtering was applied every 6 min to the previously smoothed signals.

### Anomaly detection: what, and when

Real-time total signals time series from each e-nose were analyzed to detect higher-than-normal emissions of trace gases, here termed *air quality anomalies*. For each device, three annual alarm thresholds—set at the 98^th^, 99^th^, and 99.9^th^ percentiles of the previous year’s anomaly-free data—define the yellow, orange, and red alarm levels, respectively. Exceeding the yellow threshold, 98^th^ percentile, has only a 2% chance under normal conditions and thus flags a higher-than-normal emission event. Surpassing the orange level, 99^th^ percentile, indicates a more sustained anomaly, while exceeding the red level, 99.9^th^ percentile—an event with a mere 0.1% probability—denotes a high certainty of an anomaly. During analysis, all smoothed and time-aligned readings below the yellow threshold were set to zero to exclude normal background variations.

To address the question *what*, each e-nose’s continuous total signal was compared against its location-specific alarm levels. At each timestamp, the number of devices exceeding the yellow threshold was calculated and expressed as a percentage of the total network.

To answer *when* an anomaly occurs, we applied a 30% activation rule: an event’s start time is defined as the first timestamp at which at least 30% of the network registers values above the yellow threshold, and the end time is the last timestamp meeting that same criterion. These bounds ensure a precise determination of event duration and facilitate subsequent correlation with meteorological data, thereby strengthening the overall interpretation of the anomaly.

### Source identification: where, why, and who

After anomaly detection and duration estimation, further analysis was conducted to determine *where* the anomaly occurred, *why* it happened, and *who* was likely responsible for mitigation. To address *where*, the spatial distribution of active e‑noses, those exceeding the yellow threshold during the event, was mapped using their geographic coordinates. This allowed for the assessment of whether elevated emissions were confined to a specific area or distributed across the region, supporting classification of events as either localized or widespread.

The question *why* was addressed by analyzing signal patterns across the network, evaluating correlations among sensors, and incorporating relevant meteorological data. Principal component analysis (PCA) was applied to identify patterns of similarity among sensors, while multivariate curve resolution-alternating least squares (MCR-ALS) [16–18] was used to estimate the relative contributions of different emission sources to the recorded signals.

Finally, the question *who* was approached by linking the identified source contributions and spatial signal patterns to their most likely origin. Using a combination of emission directionality, sensor locations, and wind conditions, responsibility was attributed either to sources within the monitored area or to external contributors. This step enabled the identification of relevant stakeholders for potential mitigation, including local industries or environmental authorities, depending on the inferred source location.

#### Principal component analysis (PCA) and hierarchical cluster analysis (HCA)

**Fig. 2:**
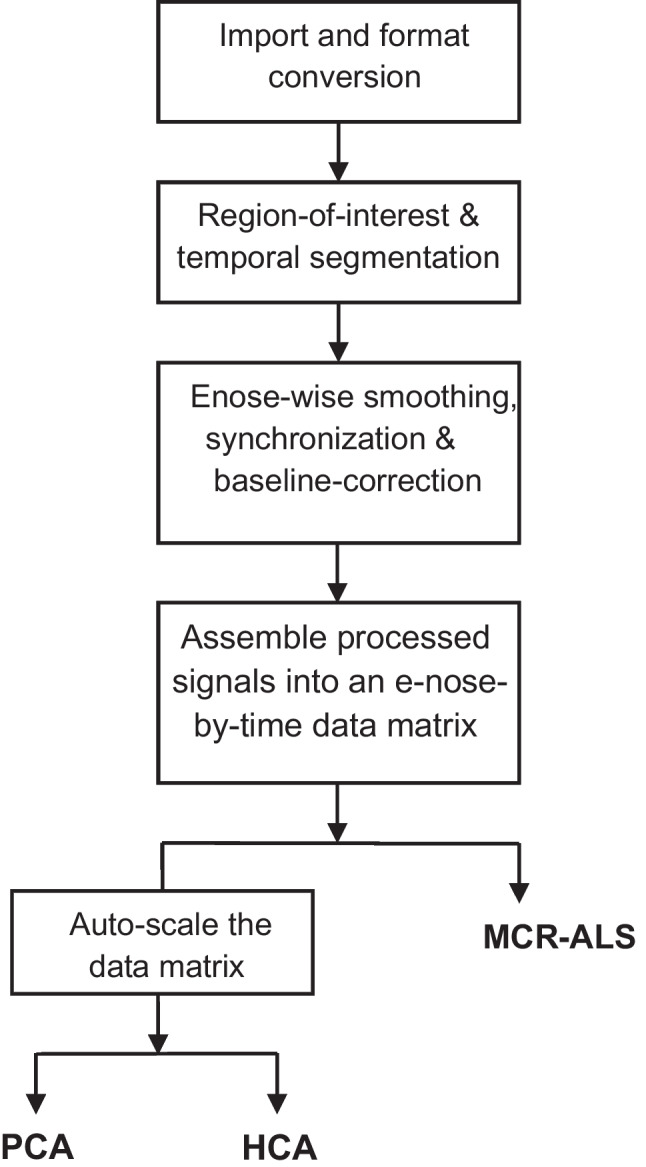
Schematic representation of the e-nose data processing and analysis workflow. Raw total signals from selected e-noses are synchronized, smoothed, and baseline-corrected, then assembled into a matrix (e-noses × time). MCR-ALS is applied to the data matrix to decompose it into contribution profiles (e-noses × sources) and source profiles (time × sources). In parallel, the data matrix was auto-scaled and the processed data served as input for PCA, which yields a score matrix (e-noses × components) and a loading matrix (time × components), and for HCA, which produces a dendrogram of sample similarities to reveal temporal and spatial emission patterns

To investigate underlying signal patterns in the network, synchronized total signal readings of individual e-noses were assembled into a matrix of dimensions (*number of e-noses* × *number of time points*), where each row represented the time series of a single e-nose. Sensor-specific offsets and scale differences across at each time point were corrected via autoscaling, ensuring equal weighting of all e-noses at every instant. A schematic representation of the data pre-processing and analysis workflow is shown in Fig. 2.

Principal component analysis (PCA) was then applied to the processed data to extract a set of orthogonal components that capture dominant patterns of variation across e-noses and time, patterns that may suggest common or distinct pollution sources. The resulting score matrix (*e-nose* × *principal components*) and loading matrix (*time points* × *principal components*) were used for further interpretation.

Hierarchical cluster analysis (HCA) [17] was also performed on the processed data matrix to group e-noses based on the dissimilarities in their signal profiles. A dendrogram based on Mahalanobis distance was generated to visualize the clustering structure, revealing groups of e-noses with similar response patterns. While these exploratory analyses do not directly identify pollution sources, they support the identification of e-nose clusters with coherent response behaviors, serving as a basis for subsequent source apportionment analysis.

#### Multivariate curve resolution-alternating least squares (MCR-ALS)

To resolve the underlying pollution sources contributing to the observed e-nose signals, the MCR-ALS [18, 19] method was applied. This method decomposes the data matrix into a set of source profiles (*components* × *time points*) and corresponding contribution profiles (*components* × *e-noses*) across the e-nose network. The number of components, representing underlying pollution sources, was initially estimated using singular value decomposition (SVD), guided by inspection of the scree plot displaying the eigenvalues of the data matrix. This selection was further supported by prior exploratory analyses, as well as the interpretability of the resulting source and contribution profiles. Initial estimates of pure components were obtained using the purest variable detection method [19], and non-negativity constraints were applied to both the source and contribution matrices to ensure physically meaningful results. This analysis allowed the resolution of overlapping emission patterns and their association with potential sources. Together with PCA and HCA, MCR-ALS forms the core of the approach used to address the *where*, *why*, and *who* components of the 5 W framework.

### Software

Data analysis was conducted using MATLAB 2021a software (MathWorks Inc., Natick, MA, USA).

## Results and discussion

This section presents the results of the proposed data-driven framework, structured around the five guiding questions—*what*, *when*, *where*, *why*, and *who*—used to detect and interpret air quality anomalies. *What* refers to identifying deviations from established baselines that indicate higher-than-normal emissions. *When* is addressed by determining the timing and duration of these events using real-time sensor data. *Where* is resolved by mapping the spatial distribution of elevated readings across the e-nose network, taking into account meteorological influences. Why is addressed by analyzing signal patterns across the network to reveal underlying emission sources and potential causes of the anomalies. Finally, who concerns the attribution of responsibility and the identification of relevant stakeholders for mitigation.

### Anomaly detection using established alarm levels

**Fig. 3:**
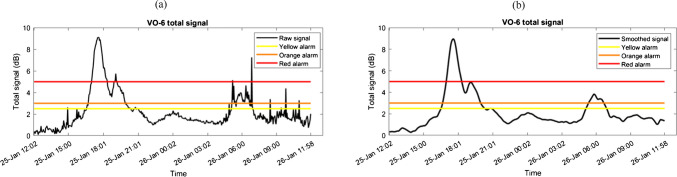
Total signal of the *VO-06* e-nose during the selected time window, shown **a** before and **b** after smoothing. Colored threshold lines indicate predefined alarm levels based on the 98^th^ (yellow), 99^th^ (orange), and 99.9^th^ (red) percentiles of the previous year’s anomaly-free data. Smoothing reduces short-term fluctuations, enhancing the robustness and reliability of anomaly detection

Anomalies were detected by comparing real-time e-nose signals to predefined alarm thresholds (98^th^, 99^th^, and 99.9^th^ percentiles), which correspond to rare signal intensities indicative of elevated pollutant concentrations. Figure 3 illustrates the raw and smoothed total signal from one of the e-noses, *VO-06*, with its yellow, orange, and red alarm levels overlaid. Two distinct anomaly periods can be identified considering the signal of this e-nose: one on January 25th between 16:30 and 20:20 and another early the following morning between 05:10 and 07:00. Signal smoothing enhanced detection reliability by increasing the signal-to-noise ratio. It is important to note that no signals were recorded from the two e-noses, *VO-01* and *BU-06*, during the study’s time window. These devices were therefore excluded from the anomaly analysis.

#### Signal intensity variations across the e-nose network

**Fig. 4:**
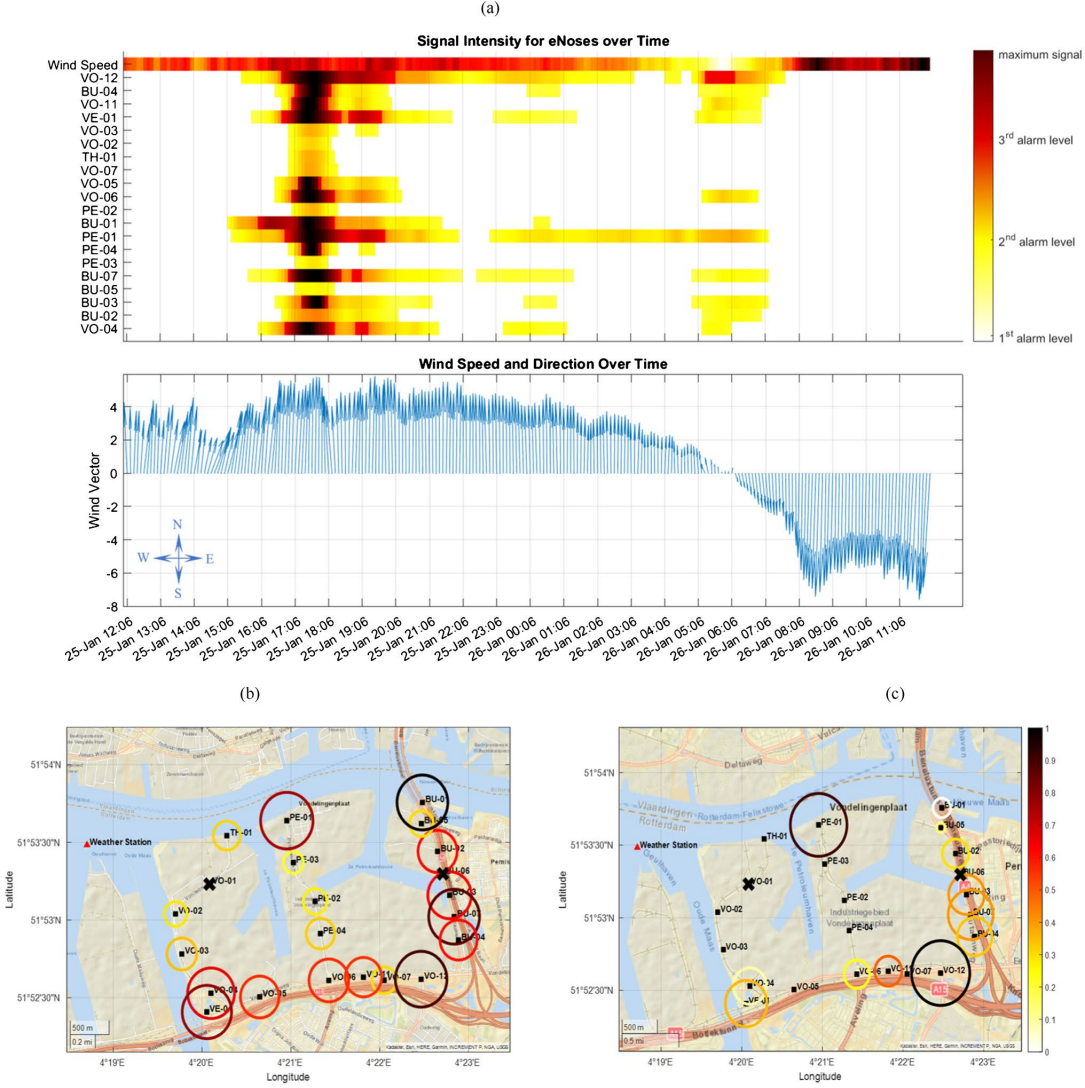
Temporal and spatial characterization of anomalies detected by e-nose network. **a** The top subplot visualizes signal intensities across all e-noses over time, using a color-coded scheme aligned with alarm levels. White represents signals below the first alarm level, while yellow, orange, and red indicate increasing intensities as higher thresholds are exceeded. The bottom subplot displays wind intensity and direction over the same period, with arrows pointing in the direction the wind is blowing toward, enabling the assessment of wind influence on detected signals. Panels **b** and **c** show the spatial distribution of signal duration and intensity during two periods: panel **b** corresponds to the first anomaly within the first 10 h, and panel **c** depicts the subsequent anomalies. Circle size represents signal duration, and color indicates average signal intensity. Two e-noses, marked with crosses on the maps, detected no signal during the selected time window

Since the anomalies detected by individual e-noses could potentially be correlated, and identifying patterns in these detections is important, a visualization tool was developed to simultaneously depict the anomalies detected by all e-noses in the target area (see Fig. 4). This tool facilitates the comparison of detection times across individual e-noses, providing a clearer and more comprehensive view of *what* occurred within the monitored region and *when*.

In the upper panel of Fig. 4a, each horizontal band represents a specific e-nose, with the x‑axis spanning the evaluation period. The lower panel illustrates wind intensity and direction over the same period, with arrows pointing in the direction the wind is blowing toward. This configuration enables assessment of wind influence on the observed signal patterns.

To effectively convey signal intensity, a color-coded scheme was employed based on the alarm levels of each e-nose and the pre-processed total signals (see Sect. 6), where values below the first alarm level are set to zero. In this visualization, white represents signals below the first level. As signal intensity increases from the first to the second alarm threshold, colors change from bright yellow to bright orange. From the second to the third alarm threshold, the colors shift from bright orange to red, with values exceeding the third threshold depicted in shades of dark red. This gradient facilitates the identification of signal patterns and highlights specific periods when certain e-noses recorded significantly stronger signals, possibly correlating with wind information.

An additional band at the top of the figure displays wind speed (scaled between 0 and 1) synchronized with the e-nose data, enabling direct comparison between signal patterns and wind speed variations.

Based on Fig. 4a, three distinct anomalies were identified within the selected time window. The first occurred between 15:00 and 21:30 on January 25th, the second between 10:30 and 1:00 the following day, and the third between 5:00 and 07:30. The first anomaly was detected by all e-noses and coincided with a notable increase in wind speed. This surge aligned with peak signal intensity across most sensors, while the wind predominantly blew from the southwest to south-southeast. In contrast, the second and third anomalies were detected by only a subset of the e-noses and exhibited weaker correlation with meteorological data. Notably, the third anomaly occurred during a period of zero wind speed; however, the e-noses continued to record high signal levels during this time. Around 6:00, the wind direction shifted sharply from south-southwest to northwest. Despite this change, the affected e-noses maintained their signal peaks, suggesting that the wind direction shift had limited influence on signal intensity.

Figure 4b and c illustrate the duration and intensity of the signals detected during the first 10 h (which include the first anomaly) and the subsequent period (which contains the last two anomalies), respectively. The radius of each circle represents the duration for which each e-nose recorded higher-than-normal emissions, while the color represents the average signal intensity. As shown in Fig. 4b, during the first anomaly, all e-noses detected the event, with those on the southern and eastern borders of the region recording stronger and longer-lasting signals. The duration and average signal intensity gradually decrease from south to north, suggesting that the source of pollution was likely located south of the target region. Given the predominant wind direction from the south, the pollution most likely originated from an external source, situated south of the area. Despite this pattern, the e-noses located along the eastern highway of the target region recorded higher-than-expected intensities and duration. Additionally, another individual e-nose, *PE-01*, detected this anomaly with a duration and intensity that do not align with this pattern, indicating that it might be responding to another source of pollution.

Figure 4c shows that the last two anomalies were detected by fewer e-noses, with weaker intensity, and were less correlated with wind speed and direction, suggesting that the source of these anomalies may lie within the monitored region. Almost all the e-noses detecting these anomalies are located along the highways, except for *PE-01*, again indicating that this e-nose may be detecting a different source of pollution. Additionally, *VO-12* detected higher-than-expected intensities and durations.

### Source identification results

To complement the visual approach and to analyze the signal patterns across the e-noses in the study area and identify similarities in their behavior over the selected time window, PCA, HCA, and MCR-ALS methods were performed. These methods provide complementary insights into the relationships between e-noses based on their signals, facilitating the identification of groups that responded similarly to emission events, and addressing the key questions of *where* the anomalies originated, *why* they occurred, and *who* could mitigate or prevent them in future.

#### PCA and HCA results

**Fig. 5:**
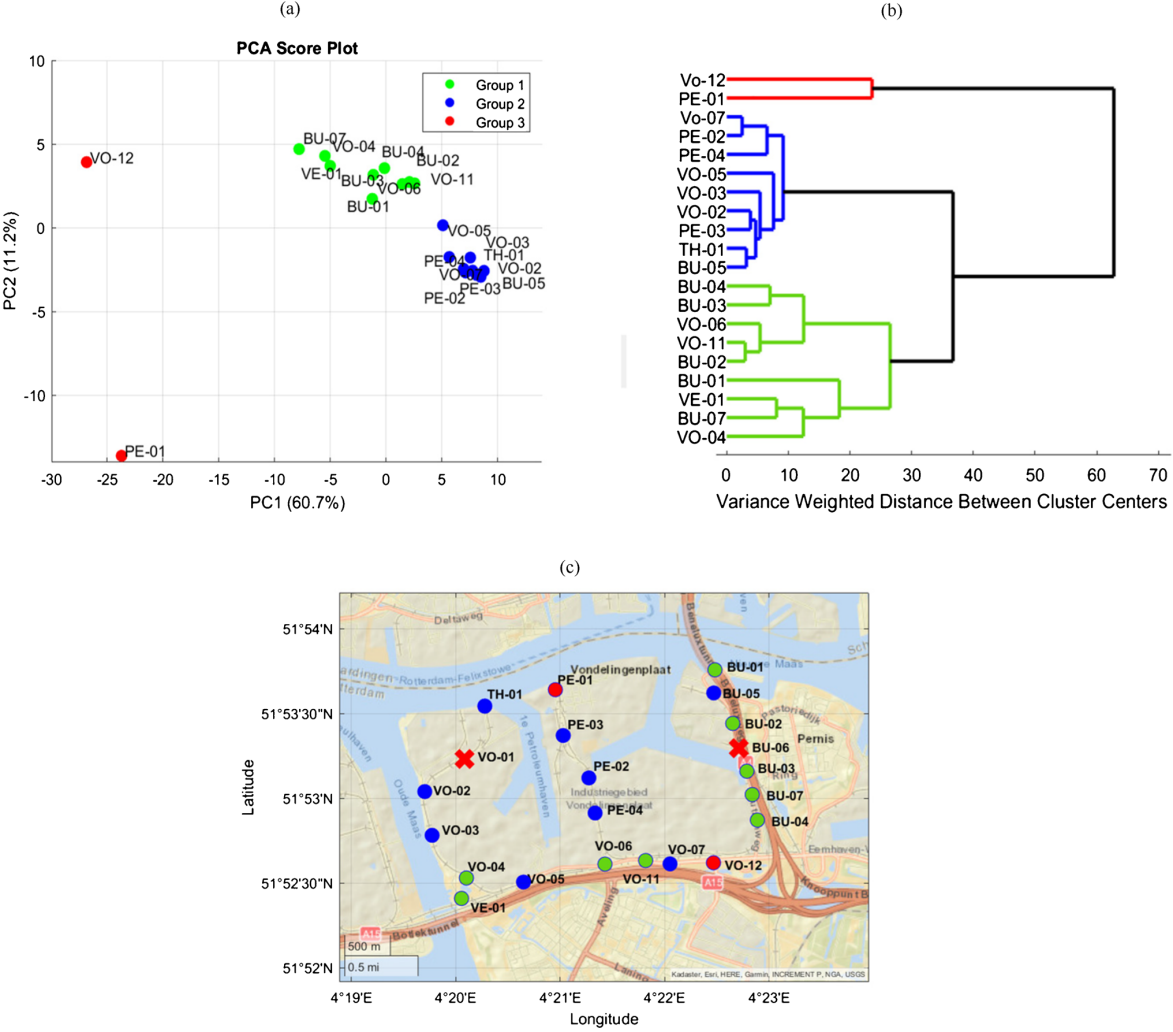
**a** PCA score plot and **b** HCA dendrogram of the processed data illustrating signal patterns across the network. Both analyses reveal three main groups, color coded for reference: Group 1 (green) clusters e-noses responding during multiple emission events. Group 2 (blue) consists of e-noses mainly responding only to the first detected emission event. Group 3 (red) includes outlier e-noses characterized by higher-than-expected signal intensity and duration. **c** Spatial distribution of the clustered e-noses highlights the geographic relevance of them: Group 1 e-noses are mostly located along highways; Group 2 e-noses are dispersed throughout the area, and Group 3 e-noses are located far from each other

The PCA score plot, Fig. 5a, visualizes the distribution of e-nose signals along the first two principal components (PCs), which together explain 72% of the variance in the dataset. Each e-nose is represented by a data point labeled with its identifier. E-noses exhibiting similar signals over time cluster closely together in this plot, while those with distinct behaviors are positioned farther apart.

In the PCA score plot, Fig. 5a, Group 3 is separated from Groups 1 and 2 along the first principal component (PC1), whereas Groups 1 and 2 are primarily separated by PC2. The corresponding loading profiles, see Electronic Supplementary Material Fig. S1, indicate that PC1 is dominated by an overall elevation in the e-nose response for Group 3, with its largest positive contributions occurring between 22:00–23:00 on 25 January and 03:00–05:00 on 26 January. In contrast, PC2 captures the distinction between Groups 1 and 2: its positive loadings reach maxima during the late‐evening intervals of 16:30–17:00 and 18:30–20:00 on 25 January and the early‐morning window of 05:00–07:30 on 26 January, whereas its unique negative peak is observed around 17:00–18:00 on 25 January. Consequently, samples with elevated signals in the positive‐loading intervals score high on PC2 and fall into Group 1, whereas samples with their strongest responses in the 17:00–18:00 window score low on PC2 and are assigned to Group 2.

To further examine the signal patterns between the e-noses, hierarchical cluster analysis was applied. The resulting dendrogram, Fig. 5b, groups the e-noses based on Mahalanobis distance, which reflects the dissimilarity between their signal profiles. E-noses with more similar signals are grouped into branches that merge at lower distances, while those with divergent patterns merge at higher distances.

While PCA and HCA do not directly identify specific emission events and their associated sources, they help differentiate groups of e-noses with similar signal behaviors over time. When combined with the earlier anomaly detection results, Fig. 4, these groupings suggest that different sets of e-noses were primarily responding to different emission events.

Group 1, which includes *VO-06* and *VO-11*, showed responses during multiple emission periods, while Group 2, consisting of e-noses such as *PE-02*, *PE-03* and *TH-01,* responded predominantly during the first detected emission event. Please refer to the “Anomaly detection using established alarm levels” section and Fig. 4 for the response patterns of these e-noses. *VO-12* and *PE-01*, which were previously shown to detect higher-than-expected emissions in both signal intensity and duration, formed a distinct Group 3 in both the PCA and HCA.

Their spatial distribution, illustrated in Fig. 5c, supports this interpretation: Group 2 is dispersed across the area, while most of the e-noses in Group1 are located along highways. This further suggests that these e-noses are likely exposed to at least two distinct pollution sources, with Group 1 likely influenced by local sources such as traffic emissions.

#### MCR-ALS results

Following the identification of three e-nose groups through PCA and HCA, with indications of three different emission patterns within the network, it became necessary to determine the origins of these emissions. The MCR-ALS method was employed to determine the number of pollution sources, their temporal profiles, and the contribution of each e-nose in detecting these sources. Three components were selected for source apportionment, indicating that the e-nose data decomposed into the variations of three distinct pollution sources. The determination of the number of components involved testing different component sizes, with the final selection based on both statistical and interpretive criteria. Various models with differing numbers of components were evaluated, and the model with three components was ultimately chosen. This decision was supported by the consistency observed in both PCA and HCA, where three distinct clusters were identified, confirming that three components provided a robust and interpretable solution. Non-negativity constraints were applied in MCR-ALS analysis to both the source profiles and contributions, ensuring physically interpretable results.

**Fig. 6:**
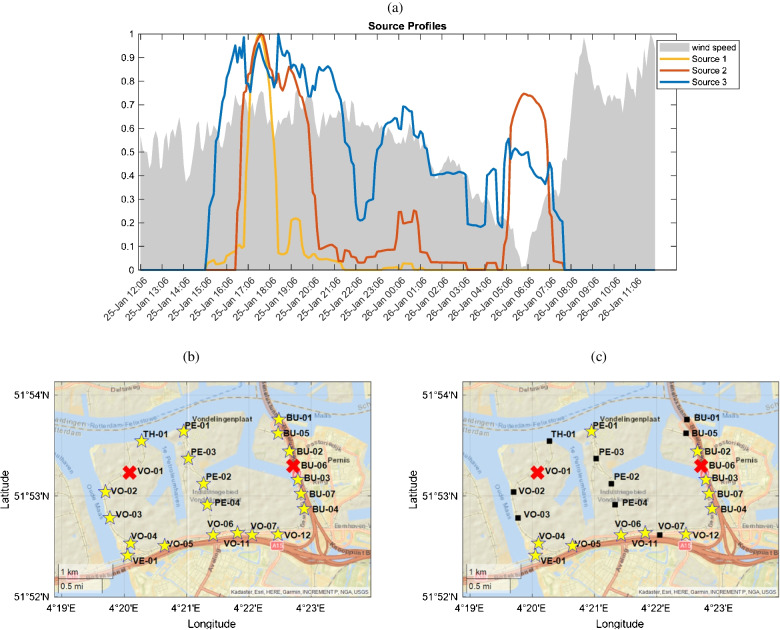
Pollution source profiles and relative contribution maps extracted by MCR-ALS. **a** Source profiles displaying the temporal variations of the three identified pollution sources, with wind speed data overlaid as a graey shaded area. **b** and **c** Spatial contribution maps for sources 1 and 2, respectively; e-noses exceeding a predefined threshold are marked with a star. Source 1 is asociated with a widespread, short-term event likely dispersed by wind; Source 2 reflects a more sustained, traffic-related emission with limited wind influence; and Source 3 is mainly detected by outlier e-noses and is characterized by sustained high signal levels

Figure 6a shows the three extracted source profiles overlaid with wind speed data, represented by a gray shaded area in the background. This visualization clarifies the relationship between the identified pollution sources and wind conditions. Each source profile is represented by a distinct color and corresponds to a specific pattern of pollutant emissions detected by the e-noses.

Analysis of the resolved contribution and source profiles revealed that they do not satisfy *data-based uniqueness* conditions [20], meaning that non-negativity constraints alone are insufficient to ensure unique solutions. In this case, there is a range of feasible solutions that obey the applied constraints and fit the data equally well. The band of feasible solutions was calculated for all profiles under soft non-negativity constraints, see Electronic Supplementary Material Fig. S2. Examination of these bands revealed that the interpretations derived from them are consistent with those obtained from the MCR-ALS analysis. For further details on the band-calculation procedure and its implications, see the Supplementary Information (SI).

With this validation in hand, we proceed to interpret each profile in terms of peak positions, assignment to specific emission sources, and their relevance to the studied network. The first source (yellow profile) displays a major peak between 17:00 and 18:00, with a minor peak around 19:15. As shown in Fig. 6b, all e-noses in the network contribute to the yellow profile, reflecting a widespread emission likely dispersed by wind across the study area. As shown in previous sections, the pollution source of this event is likely located in the southern part of the study area. Although the second peak’s intensity is much lower than the first, wind speed and direction during both peaks remain similar. This suggests that the source represented by this profile is not a stationary emission source but rather a passing plume transported and dispersed by wind. The timing of the second peak coincides with increased wind speed, indicating that residual pollutants from the initial emission were carried away, causing the observed decrease in intensity and eventual return of the source profile to baseline levels. This source profile is consistent with a known large-scale hydrogen (H_2_) plume that was reported to have passed through the region during this time window.

The second source, represented by the red profile, features two major peaks: one between 16:30 and 19:30 on January 25th, particularly intense from 16:30 to 18:00, and another from 5:00 to 7:00 the following day. A minor peak occurred between midnight and 1:00 a.m. As illustrated in Fig. 6b, this profile is strongly detected by e-noses near highways or high-traffic areas, suggesting a link to vehicular emissions. The timing of the peaks likely corresponds to rush hours in an industrial region. The shoulder of the first peak (18:00–19:30) coincides with increased wind speed, indicating wind may play a role in dispersing emissions during this time. However, the 6:00 a.m. peak was not correlated with wind speed, as wind speed drops to zero and the direction shifts during this period, see Sect. 13. This suggests that the major peaks are primarily traffic-related. Further analysis of LML data, described in the “LML data analysis” section, was conducted to reinforce and complement the insights obtained from this source.

The last source, indicated by the blue line, is primarily associated with the outlier e-noses *VO-12* and *PE-01*, which recorded signal patterns distinct from the rest of the network. As both sensors were confirmed to be functioning properly during the considered time window, the observed signals are likely due to local emissions near their locations. The temporal profile of this source shows minimal correlation with wind speed or direction, further suggesting a stationary and localized source. While the precise nature of the source remains uncertain, resolving this component is crucial in MCR-ALS analysis to remove its influence from the other sources and improve the interpretability of the results.

The results of the MCR-ALS method were consistent with findings from the PCA and HCA approaches, reinforcing the identification of pollution sources in the study area. Notably, the peaks in the PC1 loading at 22:00–23:00 on 25 January and 03:00–05:00 on 26 January, Fig. S1, align precisely with the elevated intensities of the third source profile during these intervals, reflecting the pronounced e-nose responses that were absent in the other profiles. Likewise, the two remaining source profiles reproduce key PC2 features: both exhibit elevated signals in the early-morning window (05:00–07:30) and around midnight (00:00–01:00), while also resolving the afternoon traffic peak (~ 16:30–18:00) that PCA alone could not discriminate. Together, these methods offered a detailed understanding of the sources’ origins, enabling us to distinguish between short-term, plume-driven events and longer-term emissions tied to traffic. This comprehensive analysis framework provides valuable insights into the sources’ behaviors and spatial distributions, informing strategies for targeted mitigation and prevention efforts in the future.

#### LML data analysis

The air quality monitoring data from the Local Monitoring Network (LML) was analyzed to support the interpretation of the second source profile, which was attributed to traffic emissions, and to validate the source identification. This complementary analysis helps assess whether the temporal traffic-related pattern identified by e-nose data can also be observed in the LML data of a near location, despite its different structure and nature. This dataset includes measurements of nitrogen monoxide (NO), nitrogen dioxide (NO_2_), particulate matter (PM_10_), and ozone (O_3_), which are pollutants strongly associated with traffic activities, recorded in Schiedam over the years 2021 and 2022.

To account for potential seasonal variations in pollutant levels, data from January and February of each year was extracted for analysis. Each pollutant’s data was organized into a matrix format, with rows representing individual days and columns representing hourly intervals. This structure enables a detailed temporal analysis of daily and hourly fluctuations in pollutant concentrations. By augmenting the dataset column-wise for different pollutants and conducting MCR-ALS analysis on the complete dataset, traffic-related emission patterns were extracted for direct comparison with profiles obtained from the e-nose network.

**Fig. 7:**
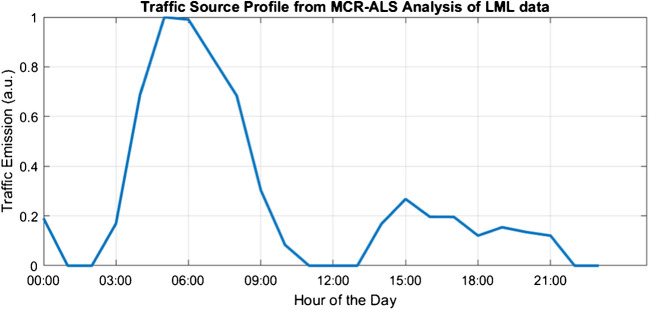
Traffic profile derived from MCR-ALS analysis of LML data. The plot illustrates the temporal variations in traffic-related pollutant emissions across a 24-h period, highlighting distinct peaks associated with morning and evening rush hours. The two distinct peaks in the morning and afternoon reflect typical rush hour patterns in a residential area near the target region and serve as a reference for comparison with Source 2 extracted from the e-nose data

To effectively capture the variation of these pollutants over time, five components were selected for the MCR-ALS analysis. This choice reflects the broader temporal range and inclusion of multiple pollutants in the LML dataset, which introduces greater variability in emission patterns in comparison to the e-nose data. Non-negativity constraints were applied to both source and contribution profiles, ensuring physically interpretable results. The analysis approach was consistent with the MCR-ALS analysis applied to the e-nose data, facilitating comparison of the results. Among these components, only the one most likely associated with traffic activities is shown in Fig. 7. The contribution profiles indicate that emissions linked to this traffic-related source peak on working days, particularly on Tuesdays and Thursdays, while weekend contributions are considerably lower.

The traffic profile extracted from the LML data recorded in Schiedam exhibits two distinct peaks: a primary peak in the early morning (~ 5:00 to ~ 9:00) and a broader peak in the late afternoon (~ 14:00 to ~ 18:00). The first and most intense peak occurs early in the day, characteristic of morning rush hour traffic. This aligns well with typical commuter patterns, reflecting increased traffic as individuals travel to work or school. The early spike in pollution levels, particularly in NO, NO_2_, and PM_10_, strongly indicates emissions from vehicle traffic associated with morning commuting.

The secondary, smaller peak in the afternoon may represent evening rush hour traffic, although it is less pronounced compared to the morning peak. This differs slightly from the e-nose traffic profile, which showed a relatively higher evening peak between 16:30 and 19:30. The lower intensity of the afternoon peak in Schiedam might reflect lower traffic density compared to Vondelingenplaat, an area with higher industrial activity, where heavy-duty vehicles likely contribute to more intense afternoon emissions.

In the e-nose traffic profile, two major peaks (in the evening and early morning) and a minor peak around midnight were identified. In contrast, the primary peak in Schiedam occurs in the morning, with a secondary peak in the afternoon. This difference in timing and intensity suggests that emissions in Schiedam are primarily driven by urban commuter traffic, while Vondelingenplaat may experience additional contributions from industrial or heavy-duty vehicle traffic, particularly in the evening.

These observations suggest that the differences in emission profiles between Schiedam and Vondelingenplaat are likely influenced by the varying nature of traffic in these areas. Schiedam’s profile may predominantly reflect commuter traffic patterns, while the dominant evening peak in Vondelingenplaat could indicate a combination of heavy-duty and industrial vehicle emissions. This interpretation provides a possible explanation for the observed discrepancies, highlighting the potential contribution of industrial activities to emissions in more industrialized regions.

## Conclusion

This study demonstrates the effectiveness of a network of e-noses, combined with PCA, HCA, and MCR-ALS analyses, in driving a high-resolution approach to air quality monitoring. The integration of these methods enabled the identification of pollution sources and provided detailed insights into their temporal and spatial patterns within the study area. Notably, the MCR-ALS results were consistent with those from PCA and HCA, reinforcing the detection of two primary emission events: a short-term event attributed to a passing plume from the southern area and a longer-term event linked to traffic emissions. The inclusion of wind data further supported these findings by highlighting the role of meteorological conditions in pollutant dispersion.

The proposed framework systematically applies the *5W* methodology by addressing *what* anomalies are, *when* and *where* they occur, *why* they are present, and *who* should take mitigation action. By enabling real-time and spatially distributed monitoring, this approach provides a proactive, data-driven method for managing air quality, informing policy decisions, and preventing potential pollution incidents.

Two key areas warrant further investigation. First, the 30% threshold, currently used heuristically to define the start and end times of emission events, requires further analysis. Future work will conduct a sensitivity study to determine the optimal threshold for pollution event detection. Second, although the framework systematically addresses the *5W* questions, it does not currently quantify the uncertainty associated with these responses. Future studies should explore appropriate uncertainty estimation techniques, such as generating confidence intervals for each 5 W element, to enhance the framework’s overall reliability.

## Supplementary Information

Below is the link to the electronic supplementary material.

ESM 1 (DOCX 286 KB)

## Data Availability

The datasets used and analyzed during the current study are available from the corresponding author on request.

## References

[1] World Health Organization. Air quality guidelines for Europe. 2nd ed. In: Publication WHO Team Air Quality, Energy and Health (AQE), Chemical Safety and Health Unit (CHE), editors. Regional Office for Europe. 2000. p. 273. https://www.who.int/publications/i/item/9789289013581.

[2] World Health Organization. Air quality guidelines global update 2005, particulate matter, ozone, nitrogen dioxide and sulfur dioxide. In: WHO Team Air Quality, Energy and Health (AQE), Environment, Climate Change and Health (ECH). 2005. p. 496. https://www.who.int/publications/i/item/WHO-SDE-PHE-OEH-06.02.

[3] Ocko IB, Hamburg SP. Climate consequences of hydrogen emissions. Atmos Chem Phys. 2022;22(14):9349–68.

[4] Webster R, Oliver MA. Geostatistics for environmental scientists. John Wiley & Sons; 2007. 10.1002/9780470517277.

[5] Gardner JW, Bartlett PN. A brief history of electronic noses. Sens Actuators, B Chem. 1994;18(1–3):210–1.

[6] Snyder EG, Watkins TH, Solomon PA, Thoma ED, Williams RW, Hagler GS, Shelow D, Hindin DA, Kilaru VJ, Preuss PW. The changing paradigm of air pollution monitoring. Environ Sci Technol. 2013;47(20):11369–77. 23980922 10.1021/es4022602

[7] Castell N, Dauge FR, Schneider P, Vogt M, Lerner U, Fishbain B, Broday D, Bartonova A. Can commercial low-cost sensor platforms contribute to air quality monitoring and exposure estimates? Environ Int. 2017;99:293–302. 28038970 10.1016/j.envint.2016.12.007

[8] NAMUR– Interessengemeinschaft Automatisierungstechnik der Prozessindustrie e.V. Technologie-roadmap „prozess-sensoren 2027+“. NAMUR – Interessengemeinschaft Automatisierungstechnik der Prozessindustrie e.V., Germany. 2021. https://www.namur.net/fileadmin/media_www/Dokumente/Roadmap_Prozesssensoren_2027.pdf.

[9] Hopke PK. Review of receptor modeling methods for source apportionment. J Air Waste Manag Assoc. 2016;66(3):237–59. 26756961 10.1080/10962247.2016.1140693

[10] Spinelle L, Gerboles M, Kok G, Persijn S, Sauerwald T. Review of portable and low-cost sensors for the ambient air monitoring of benzene and other volatile organic compounds. Sensors. 2017;17(7): 1520. 28657595 10.3390/s17071520 PMC5539520

[11] Cheng L, Meng Q-H, Lilienthal AJ, Qi P-F. Development of compact electronic noses: a review. Meas Sci Technol. 2021;32(6): 062002.

[12] Commission E (2024) Industrial Accidents and Safety. https://environment.ec.europa.eu/topics/industrial-emissions-and-safety/industrial-accidents_en. Accessed 21/01/2025.

[13] Jacobsen A, de Miranda Azevedo R, Juty N, Batista D, Coles S, Cornet R, Courtot M,Crosas M, Dumontier M, Evelo CT, Goble C, Guizzardi G, Hansen KK, Hasnain A, Hettne K, Heringa J, Hooft RW, Imming M, Jeffery KG, Kaliyaperumal R, Kersloot MG, Kirkpatrick CR, Kuhn T, Labastida I, Magagna B, McQuilton P, Meyers N, Montesanti A, vanReisen M, Rocca-Serra P, Pergl R, Sansone S-A, da Silva Santos LOB, Schneider J, Strawn G, Thompson M, Waagmeester A, Weigel T, Wilkinson MD, Willighagen EL, Wittenburg P, Roos M, Mons B, Schultes E. FAIR principles: interpretations and implementation considerations. J Data Intell. 2020;2(1–2):10–29. 10.1162/dint_r_00024.

[14] Sloan MC. Aristotle’s nicomachean ethics as the original locus for the septem circumstantiae. Class Philol. 2010;105(3):236–51.

[15] Robertson D. A note on the classical origin of “circumstances” in the medieval confessional. Stud Philol. 1946;43(1):6–14.

[16] Cleveland WS, Devlin SJ. Locally weighted regression: an approach to regression analysis by local fitting. J Am Stat Assoc. 1988;83(403):596–610.

[17] Ward JH Jr. Hierarchical grouping to optimize an objective function. J Am Stat Assoc. 1963;58(301):236–44.

[18] Tauler R, Viana M, Querol X, Alastuey A, Flight R, Wentzell PD, Hopke P. Comparison of the results obtained by four receptor modelling methods in aerosol source apportionment studies. Atmos Environ. 2009;43(26):3989–97.

[19] Jaumot J, Gargallo R, De Juan A, Tauler R. A graphical user-friendly interface for MCR-ALS: a new tool for multivariate curve resolution in MATLAB. Chemom Intell Lab Syst. 2005;76(1):101–10.

[20] Lakeh MA, Abdollahi H, Rajkó R. Predicting the uniqueness of single non-negative profiles estimated by multivariate curve resolution methods. Anal Chim Acta. 2022;1199: 339575. 35227383 10.1016/j.aca.2022.339575

