# Supplementary material for: An end-to-end data analysis framework for real-time detection and source identification of pollution events via e-nose networks
Source: Anal Bioanal Chem. 2025 Jul 24;417(25):5771–83. doi: 10.1007/s00216-025-06014-8 (PMC12528343; doi:10.1007/s00216-025-06014-8)
Supplement: Supplementary file 1 — (DOCX 286 KB) [file 216_2025_6014_MOESM1_ESM.docx]

**Analytical and Bioanalytical Chemistry**

**Supporting information**

**An end-to-end data analysis framework for real-time detection and source identification of pollution events via e-nose networks**

Mahsa Akbari Lakeh ^a^, Simon Bootsma ^b^, Ralph van Nellestijn ^b^, Gerjen H. Tinnevelt ^a^_,_ Jeroen J. Jansen ^a^

^a^ Radboud University, Institute for Molecules and Materials, (Analytical Chemistry), P.O. Box 9010, 6500 GL Nijmegen, The Netherlands

^b^ Comon Invent B.V., Burgemeestersrand 198a, 2625 NZ Delft, The Netherlands

**Figures**

| 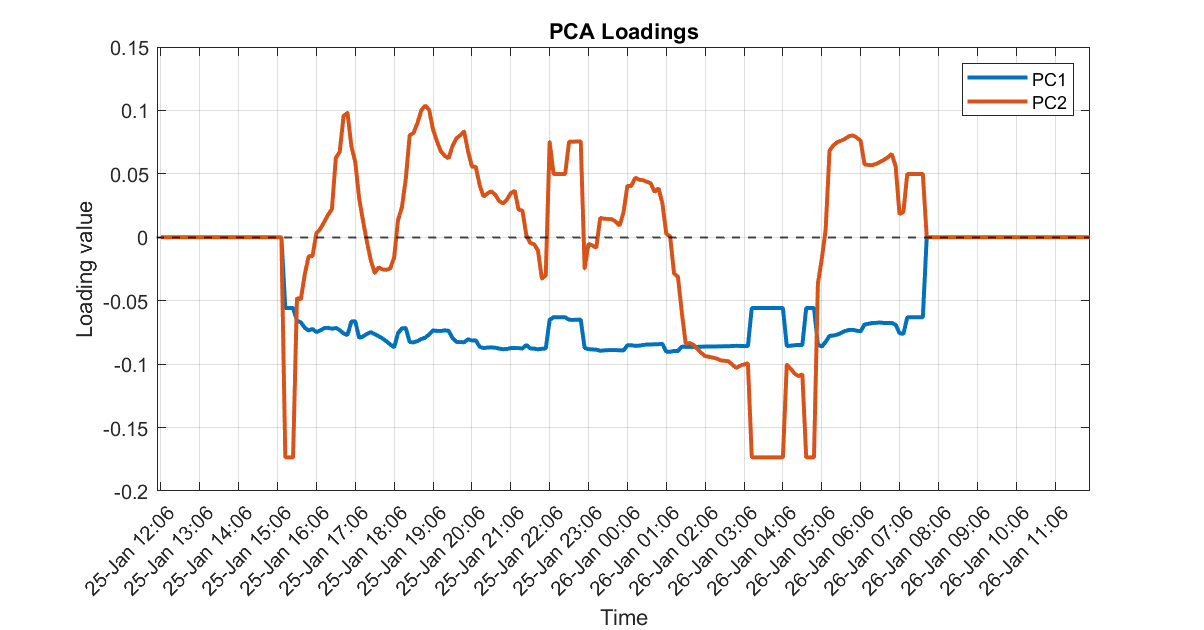 |
| --- |
| Figure S1. Loading profiles of the first two principal components extracted from the PCA of processed e-nose data. PC1 is dominated by an overall elevation in e-nose responses corresponding to Group 3, while PC2 captures time windows distinguishing Groups 1 and 2, including several positive peaks, such as one during 05:00–07:30, and a unique negative peak around 17:00–18:00 on 25 January. |

| (a)  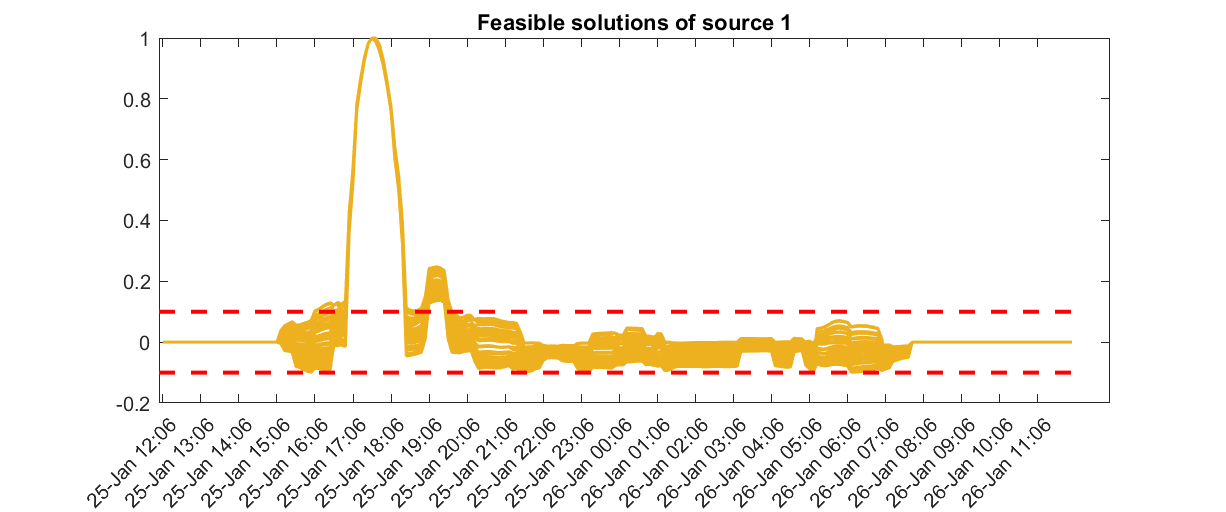 | (b)  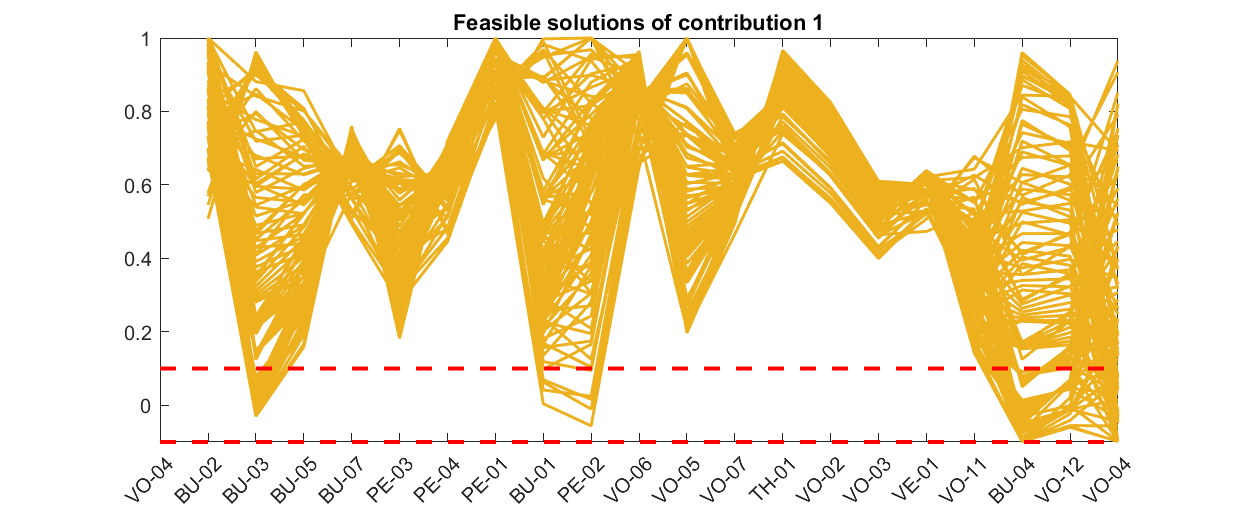 |
| --- | --- |
| (c)  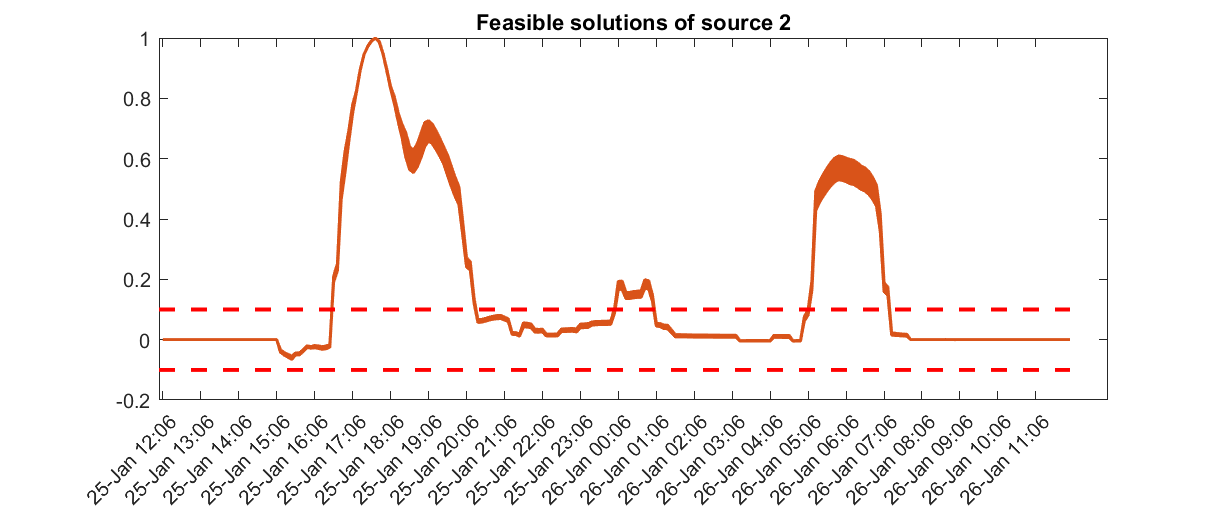 | (d)  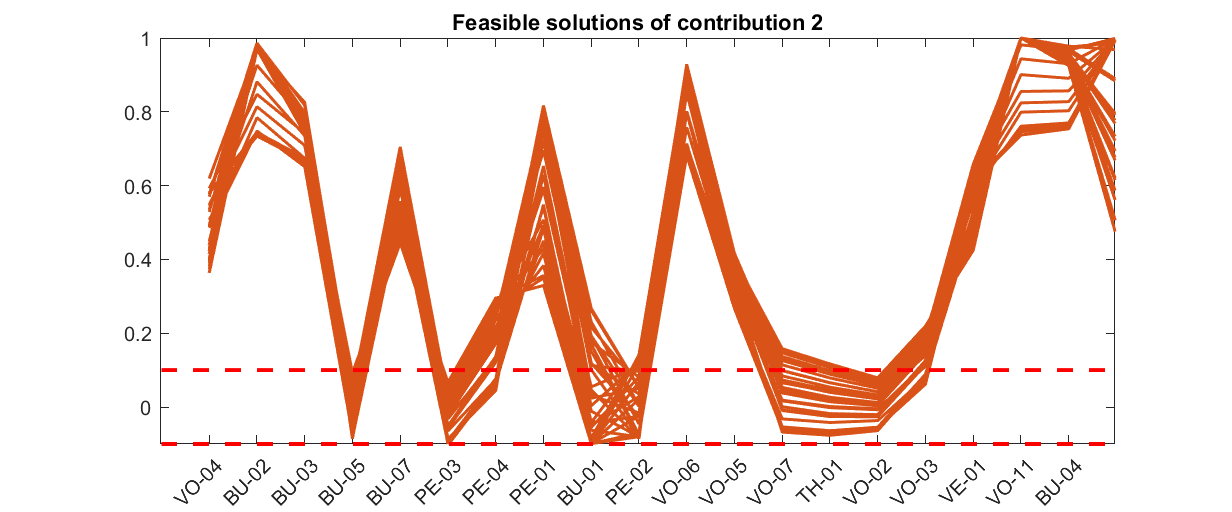 |
| (e)  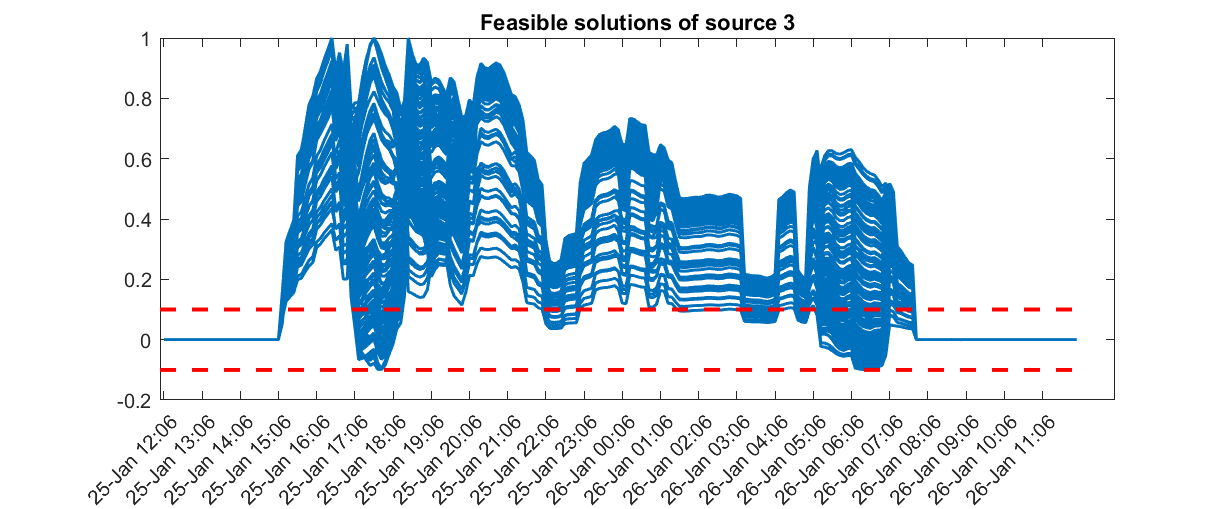 | (f)  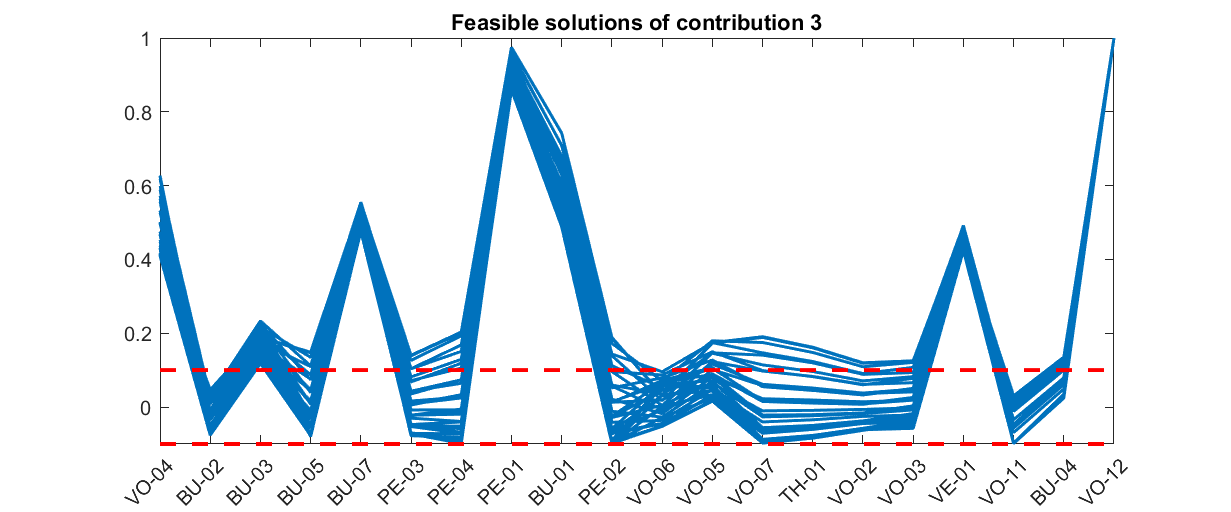 |
| **Figure S2**. Feasible solutions of the source profiles (a, c, and e) and the contribution profiles (b, d, and f) obtained using the polygon inflation algorithm with soft non-negativity constraints applied to both modes. The level of uncertainty was estimated from the initial NMF fit and used to define a significance threshold, indicated by red dashed horizontal lines. Values within this range are considered not significantly different from zero, aiding in the interpretations of the band of feasible solutions. This aids in identifying temporal patterns, e.g., duration and timing of pollution events, and in determining which e-noses significantly contribute to each source. | |

The bands of feasible solutions were obtained with the FACPACK toolbox [1] using a polygon inflation algorithm, starting from an initial estimate provided by non-negative matrix factorization (NMF). In these calculations, soft non-negativity constraints [2] were applied to obtain reliable solution bands, **Fig. S2**. In this approach, a small amount of negative values (ε) is tolerated during the calculation of the feasible solution range to enhance robustness.

The value of ε was estimated from the initial NMF solutions and quantified as the most negative entry relative to each profile’s maximum [2]. For the e‑nose data matrix, ε was estimated to be −0.01. To identify which elements in the feasible bands deviated significantly from zero, each contribution or source profile was $L_{\infty}$-normalized and subjected to a fixed threshold of 0.01 (1% of the profile maximum). Values with absolute magnitude below this threshold were considered not significantly different from zero, reflecting the data-driven level of uncertainty obtained from the initial NMF fit.

Comparison of the MCR-ALS resolved profiles with the bands of feasible solutions confirms that the key features identified in the MCR-ALS resolved profiles are preserved. Specifically, the same temporal patterns, such as the duration and timing of the pollution events can be observed across the feasible solution ranges. For instance, in the first source profile (yellow), both the resolved and feasible profiles display a major peak between 17:00 and 18:00 and a secondary peak around 19:15, suggesting consistency in detecting the underlying emission events. Likewise, the corresponding contribution profiles indicate that the same subset of e-noses are primarily associated with this source, especially when considering the applied significance threshold.

1. Sawall M, Neymeyr K (2014) A fast polygon inflation algorithm to compute the area of feasible solutions for three‐component systems. II: theoretical foundation, inverse polygon inflation, and FAC‐PACK implementation. Journal of Chemometrics 28 (8):633-644

2. Sawall M, Rahimdoust N, Kubis C, Schroeder H, Selent D, Hess D, Abdollahi H, Franke R, Boerner A, Neymeyr K (2015) Soft constraints for reducing the intrinsic rotational ambiguity of the area of feasible solutions. Chemometrics and Intelligent Laboratory Systems 149:140-150
